# Stim and Orai proteins in neuronal Ca^2+^ signaling and excitability

**DOI:** 10.3389/fncel.2015.00153

**Published:** 2015-04-24

**Authors:** Francesco Moccia, Estella Zuccolo, Teresa Soda, Franco Tanzi, Germano Guerra, Lisa Mapelli, Francesco Lodola, Egidio D’Angelo

**Affiliations:** ^1^Laboratory of General Physiology, Department of Biology and Biotechnology “Lazzaro Spallanzani”, University of PaviaPavia, Italy; ^2^Neurophysiology Unit, Department of Brain and Behavioral Sciences, University of PaviaPavia, Italy; ^3^Department of Medicine and Health Sciences, University of MoliseCampobasso, Italy; ^4^Museo Storico della Fisica e Centro di Studi e Ricerche Enrico FermiRoma, Italy; ^5^Laboratory of Molecular Cardiology, IRCCS Fondazione Salvatore MaugeriPavia, Italy; ^6^Brain Connectivity Center, C. Mondino National Neurological Institute, Fondazione IRCCS Policlinico San Matteo PaviaPavia, Italy

**Keywords:** STIM1, Orai1, STIM2, Orai2, store-operated Ca^2+^ entry, Ca^2+^ signaling, neurons

## Abstract

Stim1 and Orai1 are ubiquitous proteins that have long been known to mediate Ca^2+^ release-activated Ca^2+^ (CRAC) current (I_CRAC_) and store-operated Ca^2+^ entry (SOCE) only in non-excitable cells. SOCE is activated following the depletion of the endogenous Ca^2+^ stores, which are mainly located within the endoplasmic reticulum (ER), to replete the intracellular Ca^2+^ reservoir and engage specific Ca^2+^-dependent processes, such as proliferation, migration, cytoskeletal remodeling, and gene expression. Their paralogs, Stim2, Orai2 and Orai3, support SOCE in heterologous expression systems, but their physiological role is still obscure. Ca^2+^ inflow in neurons has long been exclusively ascribed to voltage-operated and receptor-operated channels. Nevertheless, recent work has unveiled that Stim1–2 and Orai1-2, but not Orai3, proteins are also expressed and mediate SOCE in neurons. Herein, we survey current knowledge about the neuronal distribution of Stim and Orai proteins in rodent and human brains; we further discuss that Orai2 is the main pore-forming subunit of CRAC channels in central neurons, in which it may be activated by either Stim1 or Stim2 depending on species, brain region and physiological stimuli. We examine the functions regulated by SOCE in neurons, where this pathway is activated under resting conditions to refill the ER, control spinogenesis and regulate gene transcription. Besides, we highlighted the possibility that SOCE also controls neuronal excitation and regulate synaptic plasticity. Finally, we evaluate the involvement of Stim and Orai proteins in severe neurodegenerative and neurological disorders, such as Alzheimer’s disease and epilepsy.

## Introduction

**FIGURE 1:**
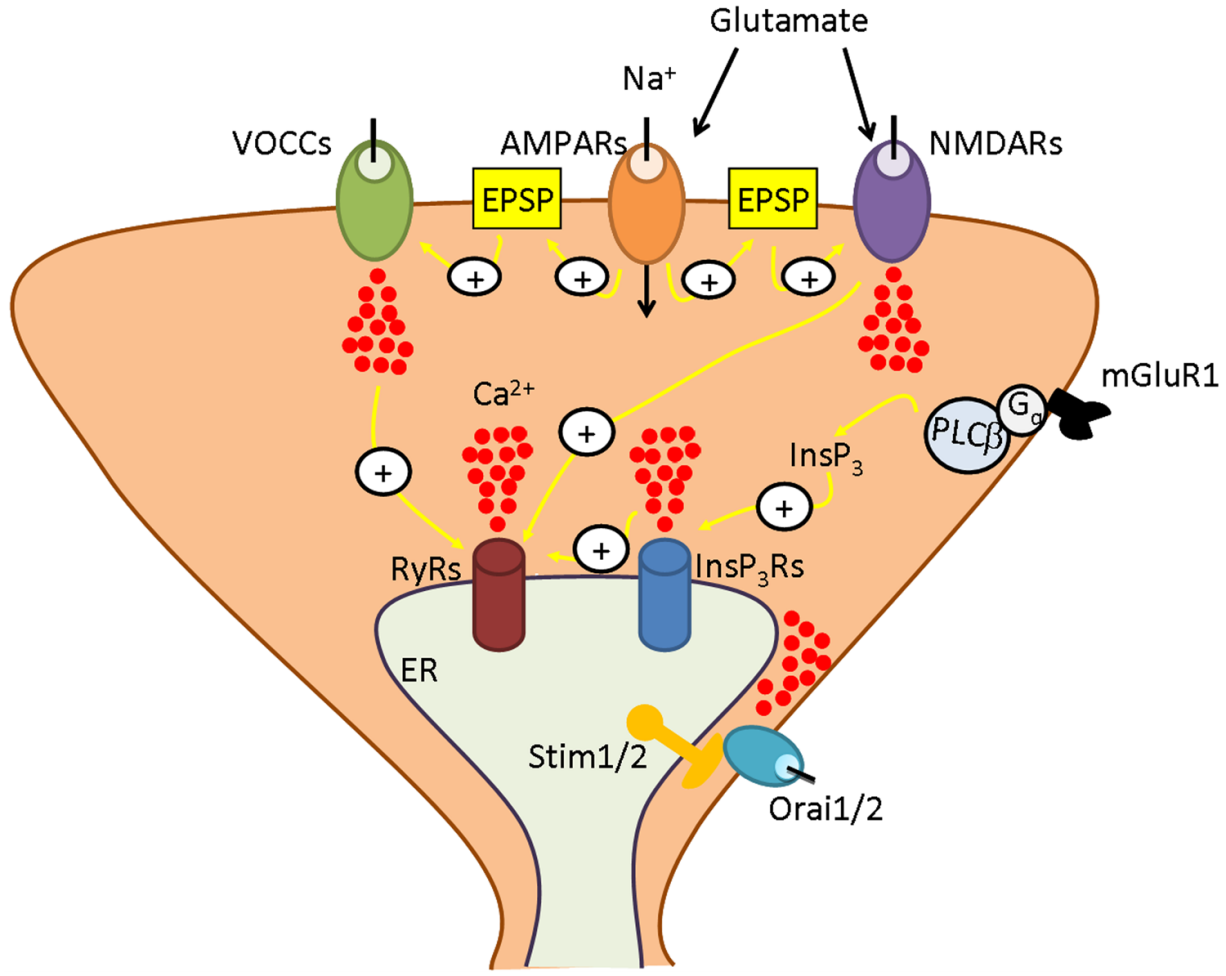
**The neuronal Ca^2+^ signalling toolkit**. Neuronal Ca^2+^ signals are shaped by the interaction between Ca^2+^ inflow from the outside and Ca^2+^ mobilization from the endoplasmic reticulum (ER), their most abundant endogenous Ca^2+^ pool. At excitatory synapses, the signaling cascade is initiated when glutamate is released into the synaptic cleft. Glutamate binds to receptor-operated channels, such as α-amino-3-hydroxy-5-methyl-4-isoxazolepropionic acid receptors (AMPARs) and *N*-methyl-D-aspartate receptors (NMDARs), and to metabotropic receptors, such as type 1 metabotropic glutamate receptors (mGluR1). AMPAR gates Na+ entry, thereby causing the excitatory postsynaptic potential (EPSP) that removes the Mg^2+^ block from NMDAR , enabling it to open in response to Glu and to mediate Ca^2+^ inflow. Moreover, the EPSP recruits an additional pathway for Ca^2+^ entry by activating voltage-operated Ca^2+^ channels (VOCCs). Outside the postsynaptic density is located mGluR1, that is coupled to PLCb by a trimeric Gq protein and, therefore, leads to inositol-1,4,5-trisphosphate (InsP_3_) synthesis. InsP_3_, in turn, induces Ca^2+^ release from ER by binding to and gating the so-called InsP_3_ receptors (InsP_3_Rs). ER-dependent Ca^2+^ discharge also involves ryanodine receptors (RyRs) which are activated by Ca2+ delivered either by adjoining InsP_3_Rs or by plasmalemmal VOCs or NMDARs according to the process of Ca^2+^-induced Ca^2+^ release (CICR). An additional route for Ca^2+^ influx is provided by store-operated Ca^2+^ entry, which is mediated by the interaction between the ER Ca^2+^-sensors, Stim1 and Stim2, and the Ca^2+^-permeable channels, Orai1 and Orai2. As more extensively illustrated in the text, depending on the species (rat, mouse, or human) and on the brain region (cortex, hippocampus, or cerebellum), Stim and Orai isoforms interact to mediate Ca^2+^ entry either in the presence or in the absence of synaptic activity to ensure adequate replenishment of ER Ca^2+^ loading and engage in Ca^2+^-sensitive decoders.

Neurons possess a highly developed Ca^2+^ machinery that delivers a multitude of Ca^2+^ signals precisely tailored at regulating specific neuronal functions (Berridge, 1998). As virtually any other cell type (Clapham, 2007; Moccia et al., 2014c), neurons use both intra- and extracellular Ca^2+^ sources which may interact to control Ca^2+^-dependent processes (Berridge, 1998). Ca^2+^ inflow from the external milieu is mediated by voltage-operated Ca^2+^ channels (VOCCs) or by receptor-operated channels (ROCs; **Figure 1**), such as the glutamate-sensitive *N*-methyl-D-aspartate receptors (NMDARs; Catterall, 2011; Paoletti et al., 2013). The main endogenous Ca^2+^ pool is provided by the endoplasmic reticulum (ER), a continuous endomembrane structure that extends from cell soma toward pre-synaptic terminals, axons, dendrites, and dendritic spines (Berridge, 1998). ER-dependent Ca^2+^ release is accomplished by inositol-1,4,5-trisphosphate (InsP_3_) receptors (InsP_3_Rs) or by ryanodine receptors (RyRs), which discharge Ca^2+^ in response to InsP_3_ and Ca^2+^ itself, respectively, according to the mechanism of Ca^2+^-induced Ca^2+^ release (CICR; Berridge, 1998; Verkhratsky, 2005; **Figure 1**). Capacitative calcium entry (CCE) or store-operated Ca^2+^ entry (SOCE) represents a peculiar mode of Ca^2+^ entry, which is activated following depletion of the ER Ca^2+^ pool in non-excitable cells (Parekh and Putney, 2005; Abdullaev et al., 2008; Sánchez-Hernández et al., 2010; Di Buduo et al., 2014; Moccia et al., 2014b). This pathway has been extensively investigated in immune cells where it is mediated by highly Ca^2+^-selective Ca^2+^ release-activated Ca^2+^ (CRAC) channels (Hogan et al., 2010; Shaw et al., 2013). The Ca^2+^ current carried by CRAC channels has been termed I_CRAC_ and is responsible for refilling the ER Ca^2+^ store after agonist-induced Ca^2+^ mobilization (Parekh and Putney, 2005; Potier and Trebak, 2008; Parekh, 2010; Moccia et al., 2012, 2014b); additionally, I_CRAC_ delivers a Ca^2+^ signal that is spatially restricted to the sub-membranal domain and recruits specific Ca^2+^-dependent decoders (Parekh and Putney, 2005; Parekh, 2010; Dragoni et al., 2011; Moccia et al., 2012). Stromal interaction molecule 1 (Stim1) is the ER Ca^2+^ sensor activating CRAC channels on the plasma membrane (PM; Roos et al., 2005; Zhang et al., 2005), whereas Orai1 is the pore forming component of CRAC channels (Feske et al., 2006; Vig et al., 2006; Yeromin et al., 2006). SOCE has long been thought to be absent or negligible in neurons (Putney, 2003), which gain easy access to the virtually infinite extracellular Ca^2+^ reservoir through VOCCs and ROCs. Nevertheless, earlier work demonstrated that a functional SOCE was present in hippocampal CA1 and CA3 pyramidal neurons (Emptage et al., 2001; Baba et al., 2003) and dentate granule cells (Baba et al., 2003). These studies showed that SOCE refills endogenous Ca^2+^ stores, governs spontaneous neurotransmitter release, and regulates both short and long-term synaptic plasticity in central nervous system (CNS). Moreover, a defective SOCE was associated to severe neurodegenerative disorders, such as Huntington’s disease (HD; Wu et al., 2011), Alzheimer’s disease (AD; Leissring et al., 2000; Yoo et al., 2000), and spongiform encephalopathies (Lazzari et al., 2011). It is, therefore, not surprising that Stim and Orai proteins have been discovered in both cultured neurons and brain sections and found to play a relevant role for synaptic transmission and higher cognitive functions (Berna-Erro et al., 2009; Klejman et al., 2009; Skibinska-Kijek et al., 2009; Keil et al., 2010; Ng et al., 2011; Steinbeck et al., 2011; Henke et al., 2013; Hartmann et al., 2014; Korkotian et al., 2014; Lalonde et al., 2014). Herein, we aim at providing a concise overview about the distribution and functions of Stim and Orai proteins in central neurons by focussing on their role in the maintenance of ER Ca^2+^ concentration ([Ca^2+^]_ER_), in the formation and maturation of dendritic spines and in gene expression. We also analyze the evidence in favor of Stim and Orai activation during synaptic stimulation and of their contribution to synaptic plasticity. Finally, we discuss their involvement in AD and other brain disorders, which hints at neuronal SOCE as a novel therapeutic target for neurodegenerative diseases.

## Molecular and Biophysical Characteristics of Stim and Orai Proteins

**FIGURE 2:**
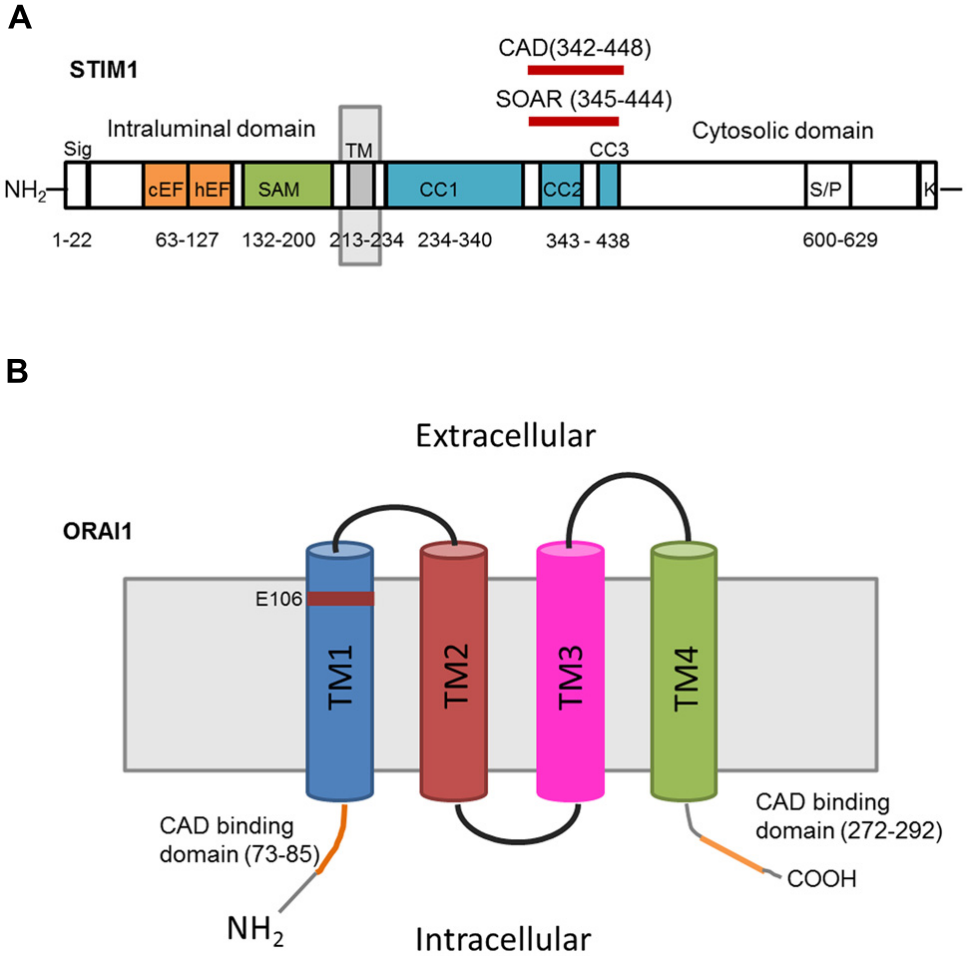
**Topology and predicted domains of Stim1 and Orai1. (A)** Stim1 comprises a signal peptide (Sig), a canonical EF-hand (cEF) domain, a hidden EF (hEF) domain, a sterile alpha motif (SAM), a transmembrane domain (TM), three coiled-coil domains (CC1, CC2, CC3), CAD, SOAR, serine/proline-rich domain (S/P), and lysine-rich domain (K-rich). **(B)** Each Orai1 monomer consists of four transmembrane domains (TM1TM4) and presents CAD binding domains in the cytosolic NH_2_ and COOH termini. E106 is the residue crucial for conferring Ca^2+^-selectivity to the channel pore.

**FIGURE 3:**
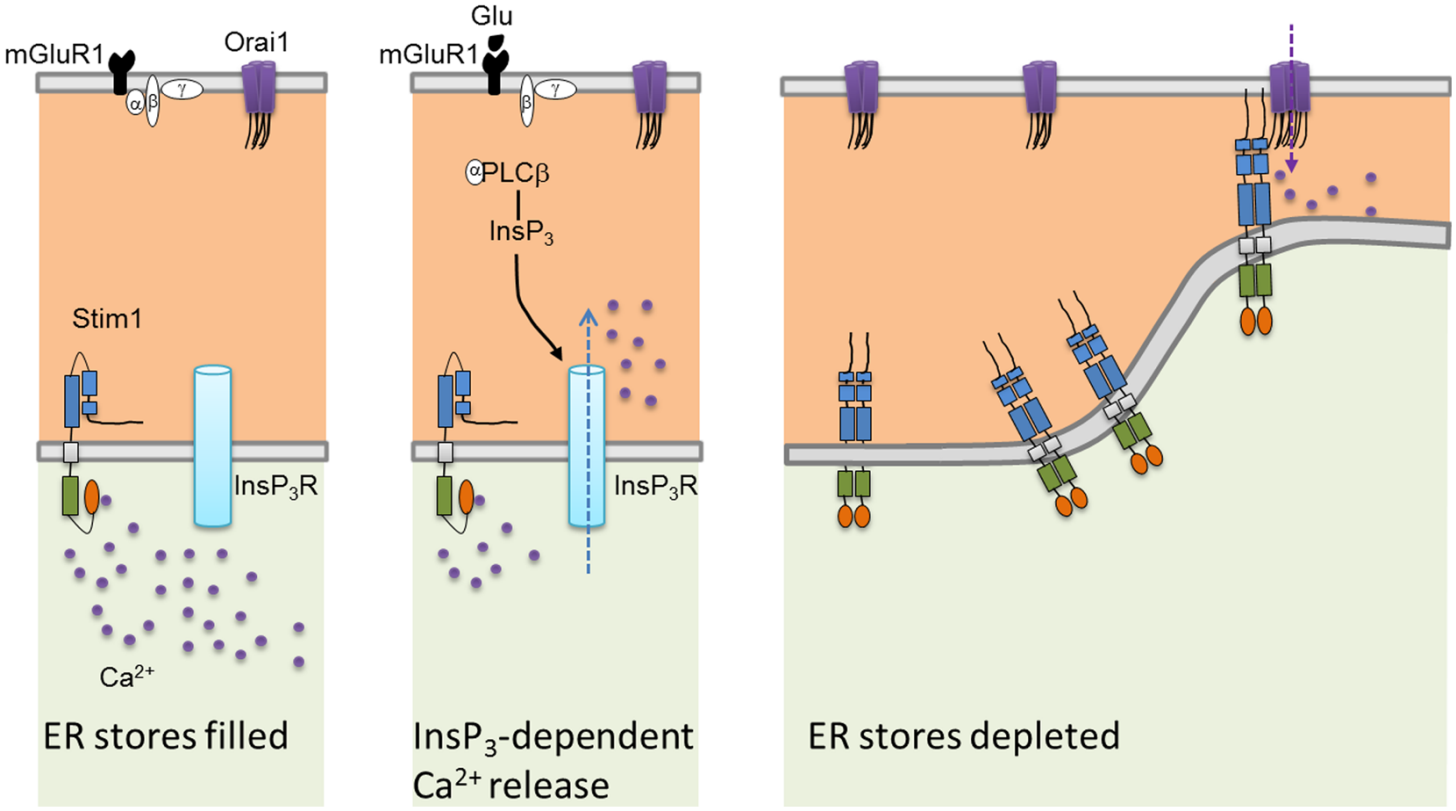
**Current model of the mechanistic coupling between Stim1 and Orai1**. In the absence of extracellular stimulation, Stim1 is uniformly distributed throughout ER membrane. Upon agonist (in this case, glutamate or Glu)-dependent PLCb activation, InsP3 is produced thereby depleting the InsP3-sensitive Ca^2+^ stores. Consequently, Ca^2+^ dissociates from Stim1 NH_2_-terminal cEF domain, resulting in SAM-mediate Stim1 oligomerization and translocation into punctate clusters in regions closely apposed to the plasma membrane. Herein, Stim1 binds to and gates Orai1 through physical interaction between, respectively, their CC domains (CC2 and CC3) and CAD binding domains, thereby activating SOCE.

Mammals have two Stim proteins (Stim1 and Stim2, sequence similarity ∼65%) and three Orai proteins (Orai1–Orai3, sequence similarity ∼89%). Stim isoforms are expressed in almost all mammalian tissues and are highly conserved from *Drosophila melanogaster* to humans. Stim1 is a type I transmembrane (TM) protein of 685 amino acids embedded either in ER membrane or on the PM where it is targeted after *N*-glycosylation of Asn131 and Asn171 (Manji et al., 2000; Williams et al., 2002). Stim1 possesses an intraluminal region of ∼22 kDa after cleavage of its signal sequence, a single TM segment, and a cytosolic domain of about 51 kDa (Shim et al., 2015; **Figure 2A**). The ER-luminal portion contains a canonical EF-hand domain (cEF), which serves as ER Ca^2+^-sensor, and a sterile alpha-motif (SAM) domain required for protein–protein interaction. A hidden, non-canonical EF-hand domain (hEF), unable to bind Ca^2+^, is also present between cEF and SAM (**Figure 2A**). The cytosolic domain comprises three coiled-coil (CC) regions (CC1-CC2-CC3), which overlap with an ezrin-radixin-moesin (ERM) motif, a serine/proline-rich (S/P) sequence and a polybasic lysine rich (K-rich) domain. In addition, the ERM domain presents crucial Orai-activating regions, which have been termed Orai1-activating small fragment (OASF), CRAC-activating domain (CAD), or Stim1–Orai1 activating region (SOAR), and include CC2 and CC3 (**Figure 1**; Shim et al., 2015; **Figure 2A**). When ER Ca^2+^ concentration falls below a threshold level due to InsP_3_R or RyRs activation, Ca^2+^ dissociates from cEF, thereby causing the unfolding of the adjacent EF-SAM domains and Stim1 multimerization (**Figure 3**). Stim1 oligomers rapidly redistribute to peripheral ER sites, termed *puncta*, in close proximity to PM, bind to and activate Orai1 (Potier and Trebak, 2008; Shim et al., 2015). Orai1, in turn, is a 33 kDa protein with a tetraspanin PM topology and cytosolic NH_2_- and COOH-tails (**Figure 2B**). Orai1 is composed of 301 amino acids, both NH_2_ and COOH termini reside in the cytoplasm, and each of them has been implicated as a critical accessory region in Orai1 activation via direct interactions with Stim1. Ca^2+^ influx is indeed gated by the physical interaction between an NH_2_-terminal domain proximal to the first TM alpha-helix of Orai1 and a COOH-terminal CC domain of the channel protein with CC2 and CC3 on Stim1 (Potier and Trebak, 2008; Shim et al., 2015). The channel pore is exclusively lined by TM1 with the residue E106 acting as crucial determinant of its high Ca^2+^-selectivity (**Figure 2B**). The crystal structure of *Drosophila* Orai1 revealed a hexameric structure, while the optimal Stim:Orai1 subunit stoichiometry seems to be 2:1 (Shim et al., 2015). Stim1 and Orai1 have clearly been established as the building blocks of SOCE. Stim1- and Orai1-mediated Ca^2+^ current displays biophysical features similar to those of the I_CRAC_ recorded in hematopoietic cells (Prakriya, 2009), i.e., non-voltage activation, strong inward rectification, reversal potential (E_rev_) > +60 mV, permeability to Ca^2+^, but not to other monovalent cations, fast and slow Ca^2+^-dependent inactivation, and a single-channel conductance in the order of femtosiemens (fS; **Table 1**; Parekh and Putney, 2005; DeHaven et al., 2007; Lis et al., 2007; Parekh, 2010). Albeit the single channel conductance of Orai1 is ∼1000 fold lower than VOCCs, the I_CRAC_ is exclusively carried by Ca^2+^ and engenders membrane-restricted Ca^2+^ microdomains where [Ca^2+^]_i_ reaches levels orders of magnitude higher than those achieved in the bulk cytoplasm (Parekh, 2010, 2011). This enables SOCE to regulate a multitude of Ca^2+^-dependent effectors that already reside within a few nanometer of the channel pore or are brought nearby upon store depletion, thereby allowing the formation of novel membrane-delimited signaling complexes (Kar et al., 2014). In addition to refilling peripheral ER juxtaposed to PM, the I_CRAC_ controls cell functions as diverse as nitric oxide (Berra-Romani et al., 2013) and arachidonic acid (Chang and Parekh, 2004) production, gene expression (Dragoni et al., 2011; Kar et al., 2012), cell cycle progression (Courjaret and Machaca, 2012; Moccia et al., 2012), mitochondrial Ca^2+^ uptake and bioenergetics(Landolfi et al., 1998; Lodola et al., 2012), exocytosis (Fanger et al., 1995), and programmed cell death or apoptosis (Dubois et al., 2014).

**Table 1:** Biophysical properties of Orai1, Orai2, and Orai3.

	Orai1	Orai2	Orai3
Store-operated	Yes	Yes	Yes
E_rev_	> +60 mV	> +60 mV	> +60 mV
I–V relationship	Inward rectification	Inward rectification	Inward rectification
PCa^2+^/Na^+^	>1,000	>1,000	>1,000
Monovalent permation in divalent-free solution	Moderate	Moderate	Strong
Single-channel conductance	9–24 fS in 2-10 mM Ca^2+^	ND: likely in the fS range	ND: likely in the fS range
Fast Ca^2+^-dependent inactivation	Moderate	Moderate	Strong
Slow Ca^2+^-dependent inactivation	Strong	None	None

*E_rev_, reversal potential; PCa^2+^/PNa^+^, Ca^2+^/Na^+^ permeability ratio; I–V relationship, current-to-voltage relationship; ND, not determined. Data obtained from DeHaven et al. (2007), Lis et al. (2007), Hoth and Niemeyer (2013).*

A second Stim isoform, predominantly present in neurons, has been identified in mammals (Schuhmann et al., 2010). Stim2 has an open reading frame of 833 amino acids and presents specific homologous regions to Stim1 within both luminal and cytosolic domains, although it does not localize on the PM. The regions that present greater homology between the two proteins include the previously mentioned SAM domain, the EF-hand motif, the three CC domains, two cysteine residues within the luminal domains and the K-rich domain altogether with the S/P-rich sequence. Furthermore, both Stim1 and Stim2 possess a single-pass TM region (Hoth and Niemeyer, 2013). However, Stim1 contains a 22-aminoacid ER-signal peptide sequence at the NH_2_-terminus, while Stim2 requires a longer ER-localization peptide (i.e., residues 1–101; Graham et al., 2011; Hoth and Niemeyer, 2013). Moreover, Stim2 displays a lower Ca^2+^-sensing affinity as related to Stim1 by virtue of three amino acid substitutions in cEF. This feature renders Stim2 able to detect smaller decreases in intraluminal Ca^2+^ with an EC_50_ of 406 μM compared to 210 μM for Stim1 (Hoth and Niemeyer, 2013). As a consequence, while Stim1 triggers SOCE during extracellular stimulation, Stim2 gates constitutive Ca^2+^ inflow in quiescent cells (Brandman et al., 2007). Two additional homologs of Orai1 gene, Orai2 and Orai3, were found in mammals with Orai3 apparently evolving directly from Orai1 rather than Orai2. Recent reviews have detailed the structural and biophysical features of these isoforms (Shuttleworth, 2012; Hoth and Niemeyer, 2013). Although the three Orai proteins display divergent NH_2_-terminal sequences, the amino acid alignment evidenced a near complete conservation within the pore-lining TM1 region, including an identical selectivity filter corresponding to E106 in Orai1. However, simple examination of the overall sequences reveals several regions where there are clear differences. Interestingly, both Orai2 and Orai3 lack the extracellular consensus site for glycosylation. Furthermore, Orai3 has the longest extracellular loop between TM3 and TM4 (21% sequence identity), and both Orai2 and Orai3 lack the ∼18 amino acid NH_2_-terminal hydrophilic stretch of Orai1, which together with the nearby Orai1-specific proline-rich domain mediate reactivation after fast Ca^2+^-dependent inactivation (Shuttleworth, 2012; Hoth and Niemeyer, 2013). Both Orai2 and Orai3 form CRAC channel when overexpressed with Stim1 (DeHaven et al., 2007; Gwack et al., 2007; Lis et al., 2007), but their physiological function remains unclear. Recent evidence, however, suggested that Orai3 mediates the I_CRAC_ under pathological conditions, such as cancer and cardiac failure (Motiani et al., 2013; Saliba et al., 2015). The pore of both Orai2 and Orai3 is selective for Ca^2+^ over Na^+^ and shows the typically inwardly rectifying current-voltage relationship as observed for the endogenous I_CRAC_ (Shuttleworth, 2012; Hoth and Niemeyer, 2013). Interestingly, fast Ca^2+^-dependent inactivation is very prominent in Orai3 compared to Orai1 and Orai2, whereas slow Ca^2+^-dependent inactivation is modest. Moreover, all three Orai channels are potentiated by divalent cations with a predilection for Ca^2+^ over Ba^2+^ and Mg^2+^. These differences were smaller for Orai3 rather than for both other Orai channels (Shuttleworth, 2012; Hoth and Niemeyer, 2013; **Table 1**). Furthermore, Orai3 can be activated by 2-aminoethoxydiphenyl borate (2-APB), which in turn inhibits both Orai1 and Orai2 (Lis et al., 2007; Moccia et al., 2014a). 2-ABP-induced Orai3 activation is, however, independent on Stim1 (Yamashita et al., 2011).

## Stim and Orai Distribution in Rodent and Human Brain Neurons

Stim1 and Stim2, as well as Orai1 and Orai2, are widely expressed in rodent and human brains (Dziadek and Johnstone, 2007; Gross et al., 2007; Gwack et al., 2007; Wissenbach et al., 2007; Berna-Erro et al., 2009; Gruszczynska-Biegala et al., 2011; Steinbeck et al., 2011; Hartmann et al., 2014). Stim1 and Stim2 proteins are uniformly distributed throughout mouse brain. Stim1 reaches its higher level of expression in the molecular layer of the cerebellum, albeit it is not more abundant than Stim2 in this region (**Table 2**; Klejman et al., 2009; Skibinska-Kijek et al., 2009; Hartmann et al., 2014). Stim2, in turn, is the predominant isoform in mouse hippocampus (**Table 2**; Berna-Erro et al., 2009; Skibinska-Kijek et al., 2009), while the levels of Stim1 and Stim2 are similar in cortex, thalamus, and amygdala (**Table 2**; Skibinska-Kijek et al., 2009; Hartmann et al., 2014). A careful inspection of the regional distribution of Stim proteins in rat brains is still lacking, yet, Stim2 mRNA is about twofold more abundant than Stim1 in rat cortical and hippocampal neurons (Gruszczynska-Biegala et al., 2011). Finally, Stim1 and Stim2 are also present in human brain and display their highest expression levels in cortex and hippocampus (**Table 3**; Steinbeck et al., 2011). At sub-cellular level, Stim1 and Stim2 reside in the ER and are more abundant in the cell body and in primary and secondary dendrites of mouse cortical, hippocampal and Purkinje neurons (Klejman et al., 2009; Skibinska-Kijek et al., 2009; Sun et al., 2014). Likewise, ER-resident Stim1 is enriched at the post-synaptic densities in rat hippocampal neurons, albeit it has also been identified on the PM (Keil et al., 2010), as originally described in (Williams et al., 2001). Finally, Stim1 expression increases during neuronal development *in vitro* and reaches relatively high and stable levels in mature neurons (Keil et al., 2010). The regional distribution of Orai proteins has been evaluated only in mouse, in which Orai1 is uniformly distributed throughout all the brain (Klejman et al., 2009), albeit the overall expression of Orai2 is significantly higher and Orai3 is absent (Berna-Erro et al., 2009). Similar to Stim1, Orai1 is subcellularly localized in the soma and primary dendrites of mouse neurons (see below; Klejman et al., 2009). Therefore, Stim and Orai proteins display a species- and region-dependent pattern of neuronal expression, which could render them capable of accomplishing specific tasks during cognitive processes.

**Table 2:** Distribution of Stim and Orai in mouse brain.

SOCE component	mRNA/protein	Species	Cerebellum	Thalamus	Hippocampus	Cortex	Amygdala
Stim1	Protein	Mouse	++	+	+	+	+
Stim2	Protein	Mouse	++	+	+++	++	++
Stim1/Stim2	Protein	Mouse	=	=	<1	=	=
Orai1	mRNA	Mouse	+	+	+	+	+
Orai2	ND	ND	ND	ND	ND	ND	ND
Orai3	ND	ND	ND	ND	ND	ND	ND

*Where available, data about protein expression were added. SOCE, store-operated Ca^2+^ entry. Data obtained from Klejman et al. (2009) and Skibinska-Kijek et al. (2009).*

**Table 3:** Distribution of Stim and Orai transcripts in human brain.

Protein	Species	Cerebellum	Thalamus	Hippocampus	Cortex	Amygdala
Stim1	Human	+	+	++	++	+
Stim2	Human	+	+	++	++	+

*ND, not determined. Data obtained from Steinbeck et al. (2011). There are no data available regarding Stim1/Stim2 ratio and Orai1-3 expression.*

## Evidence about Stim- and Orai-Mediated Ca^2+^ Entry in Brain Neurons

The interaction of the diverse Stim and Orai isoforms has been investigated in primary cultures of neurons lacking either of them (through knocked out mice or by the use of specifically targeted siRNAs) or over-expressing Orai1 and/or yellow fluorescent protein (YFP) or enhanced green fluorescent protein (EGFP)-tagged Stim proteins. These experiments have been conducted on both mouse and rat neurons. Co-expressed Orai1 and Stim1 (both in its YFP and GFP tagged forms) are evenly distributed in the soma, primary dendrites and post-synaptic dendritic spines of mouse cortical neurons, thereby confirming the localization of the endogenous proteins (Klejman et al., 2009; Ng et al., 2011). The pharmacological depletion of the ER Ca^2+^ reservoir with thapsigargin, a selective SERCA inhibitor, causes both Orai1 and Stim1 to redistribute and co-localize into *puncta*-like clusters (Klejman et al., 2009; Ng et al., 2011), as observed in non-excitable cells (Parekh, 2010; Moccia et al., 2012; Shim et al., 2015). Moreover, thapsigargin-induced Ca^2+^ release elicits a robust Ca^2+^ inflow in post-synaptic dendrites (Ng et al., 2011). Surprisingly, the physiological stimulation of type I metabotropic glutamate receptors (mGluRs) and of muscarinic receptors induces dendritic Ca^2+^ release and Ca^2+^ inflow in mouse cortical neurons, but does not elicit the formation of Stim1 *puncta*. However, this treatment reduces Stim1 mobility, which is compatible with Stim1 clusterization within post-synaptic spines (Ng et al., 2011). Although Stim1 and Orai1 co-localize upon ER depletion, they do not mediate SOCE in the mouse cortex. Accordingly, SOCE is unaffected by the genetic deletion of Stim1 and Orai1; conversely, it is absent in neurons from Stim2-deficient mice (Berna-Erro et al., 2009). Likewise, Stim2 is essential to induce SOCE in mouse hippocampal neurons (Sun et al., 2014), in which it is the most abundant isoform. These studies imply that Stim2 regulates SOCE by coupling to Orai2 in mouse cortex and hippocampus, as recently demonstrated in mouse dendritic cells (Bandyopadhyay et al., 2011). This model is supported by the lack of Orai3 expression in mouse brain, but future experiments are mandatory to assess whether Orai2 knock down suppresses SOCE in mouse cortical and hippocampal neurons. SOCE is sustained by an alternative molecular machinery in mouse cerebellum: herein, SOCE is absent in Purkinje neurons lacking Stim1 and Orai2, while it is not affected by Orai1 knockdown (Hartmann et al., 2014). Overall, these findings suggest that Orai2 provides the pore-forming subunit of CRAC channels in mouse neurons and is regulated by Stim1 in cerebellum and by Stim2 in cortex and hippocampus. This model is consistent with the fact that Stim1 and Stim2 are the most important functional isoforms in mouse cerebellum and hippocampus, respectively. The data available regarding the molecular composition of SOCE in mouse neurons have been summarized in **Table 4**.

**Table 4:** The molecular components of store-operated Ca^2+^ entry in different species and brain regions.

Species	Brain region	SOCE machinery	Reference
Mouse	Cortex	SOCE is mediated by Stim2 and, presumably, Orai2; it is present in Stim1 and Orai1-deficient neurons	Berna-Erro et al. (2009)
	Hippocampus	SOCE is mediated by Stim2 and, presumably, Orai2	Sun et al. (2014)
	Cerebellum	SOCE is mediated by Stim1 and Orai2; it is present in Orai1-deficient neurons	Hartmann et al. (2014)
Rat	Cortex and hippocampus	SOCE is triggered by either Stim1 (when is activated by massive store depletion) or Stim2 (basal Ca^2+^ entry); Orai1 is the channel pore subunit in both cases	Gruszczynska-Biegala et al. (2011)

The scenario is different in rat cortex and hippocampus, which clearly show higher levels of Stim2 as compared to Stim1. Ca^2+^ store depletion with thapsigargin reversibly enhances the association of endogenous Stim1 and Stim2 with the PM in cortical neurons; however, when the cells are co-transfected with either Stim1 and Orai1 or Stim2 and Orai1, this treatment increases the number of Stim1–Orai1 *puncta* more than nine-fold, while it does not significantly stimulate Stim2 redistribution into sub-membranal clusters (Gruszczynska-Biegala et al., 2011). Similarly, Stim1 rapidly relocates from the bulk ER to the periphery in both somatic and dendritic compartments of hippocampal neurons in response to thapsigargin (Keil et al., 2010). These data indicate that Stim1, but not Stim2, is activated following massive emptying of the ER Ca^2+^ reservoir: in other words, Stim1 is predicted to sustain SOCE during heavy extracellular stimulation in rat neurons. Conversely, Stim2 is activated and aggregates into discrete *puncta* in the absence of extracellular Ca^2+^, an artificial condition which leads to the progressive depletion of the ER Ca^2+^ reservoir and recruitment of a constitutive Ca^2+^ entry pathway to compensate Ca^2+^ leakage into the external milieu (Gruszczynska-Biegala et al., 2011). Therefore, Stim2 fulfills the double function to regulate resting Ca^2+^ inflow and maintain ER Ca^2+^ levels in rat neurons. Consistent with these observations, co-expressing Orai1 with Stim1 causes a statistically relevant elevation in SOCE, whereas transfecting the neurons with Orai1 and Stim2 enhances both constitutive Ca^2+^ influx and resting Ca^2+^ levels (Gruszczynska-Biegala et al., 2011). Likewise, a recent study from the same group has demonstrated that a small drop in ER Ca^2+^ levels induces the formation of hetero-complexes between endogenous Stim2 and Orai1 proteins in primary cortical neurons, thereby refilling the intracellular Ca^2+^ stores (Gruszczynska-Biegala and Kuznicki, 2013). Thus, Stim2 and Stim1 play distinct roles in Ca^2+^ homeostasis in rat neurons by converging on Orai1 to mediate SOCE, respectively, in response to extracellular stimulation and under resting conditions (**Table 4**).

## SOCE Controls Spine Morphology in Brain Neurons

The role of Stim1- and Stim2-mediated SOCE in brain neurons has just begun to be deciphered. Available information regards the involvement of neuronal SOCE in the control of spine architecture, ER Ca^2+^ content, and gene expression in mouse. Post-synaptic dendritic spines are the primary recipient of excitatory inputs in most central neurons and may be broadly classified into three groups depending on their morphology: mushroom spines, thin spines, and stubby spines (Sala and Segal, 2014). Long-term potentiation (LTP) leads to a structural shift from thin to mushroom spines, while long-term depression (LTD) causes spine retraction or shrinkage (Bourne and Harris, 2007). It has, therefore, been suggested that thin spines are “learning spines” that function during memory formation, while mushroom spines serve as “memory spines” that store the memory of past synaptic activity (Bourne and Harris, 2007; Matsuzaki, 2007). As mentioned above, Stim1, Stim2, and Orai1 proteins have been identified in dendritic spines in mouse cortical, hippocampal, and Purkinje neurons (Klejman et al., 2009; Skibinska-Kijek et al., 2009; Hartmann et al., 2014; Korkotian et al., 2014; Sun et al., 2014). Stim1 and Orai1 are preferentially located to mushroom spines by synaptopodin (SP), an actin-binding protein that controls both Ca^2+^ release and SOCE in these compartments (Korkotian et al., 2014; Segal and Korkotian, 2014). SP-dependent Ca^2+^ signaling controls spine head enlargement during LTP in the CA1 region of the hippocampus and drives important cognitive processes, such as spatial learning (Deller et al., 2003; Korkotian et al., 2014). Particularly, SP potentiates glutamate-induced Ca^2+^ release in dendritic spines of cultured hippocampal neurons (Vlachos et al., 2009). SP has recently been postulated to regulate activity-dependent Ca^2+^ signals by recruiting Stim1 and Orai1 to the post-synaptic density (Korkotian et al., 2014; Segal and Korkotian, 2014). However, there is no evidence that the genetic deletion of Stim1 and/or Orai1 interferes with SP-dependent increase in the Ca^2+^ response to glutamate. Moreover, it is not clear whether Stim1 and Orai1 mediate SOCE in mouse hippocampus at all. It is conceivable that Stim1 and Orai1 regulate processes other than the I_CRAC_ in this context by interacting with additional molecular partners. For instance, Stim1 is coupled to Ras homolog gene family, member A (RhoA) activation and stress fiber formation in microvascular endothelial cells (Shinde et al., 2013). Future work might assess whether Stim1 directly drives F-actin polymerization during spine morphogenesis in mouse hippocampus with or without Orai1 intervention. The consequent expansion of spine-associated ER could underpin the reported increase in glutamate-induced Ca^2+^ signals or regulate synaptically triggered biochemical cascades. Alternatively, Stim1 might be recruited by SP to the post-synaptic density to activate transient receptor potential (TRP) Canonical 3 (TRPC3), as shown in mouse cerebellar Purkinje neurons (Hartmann et al., 2014). TRPC3 presents a sizeable Ca^2+^ permeability and could contribute to the overall increase in [Ca^2+^]_i_ elicited by glutamate in dendritic spines (Hartmann et al., 2014). Finally, Stim1 could prevent cytotoxic Ca^2+^ overload by inhibiting voltage-dependent Ca^2+^ entry with or without Orai1 contribution, as extensively illustrated below (see paragraph entitled “Stim1 interaction with voltage-operated Ca^2+^ channels”). It is, therefore, clear that more work is required to fully understand the structural and functional relationships between SP, Stim1 and Orai1. While the role of Stim1 and Orai1 in the control of spine architecture is still uncertain, Stim2-mediated SOCE maintains mushroom spine structure in mouse hippocampus both *in vitro* and *in vivo* (Sun et al., 2014). Continuous Ca^2+^ inflow via Stim2-regulated store-operated channels engages Ca^2+^/calmodulin-dependent protein kinase II (CaMKII) to support long-term stabilization of mushroom spines even in the absence of synaptic activity (Sun et al., 2014). This finding is consistent with the notion that Stim2 controls SOCE in mouse hippocampus (see above); however, the finding that this pathway may also be activated under resting conditions, i.e., in non-firing neurons, deserves further consideration.

## Constitutive SOCE Maintains ER Ca^**2+**^ Levels in Brain Neurons

Ca^2+^ influx into dendritic spines is normally attributed to VOCCs and ROCs (Catterall, 2011; Paoletti et al., 2013), which operate during synaptic transmission, but are silent at rest (Hooper et al., 2014). It has long been known that neuronal ER Ca^2+^ store is partially emptied even in quiescent neurons and is replenished by a voltage-independent Ca^2+^ entry pathway that is active at subthreshold membrane potentials (Garaschuk et al., 1997; Usachev and Thayer, 1997; Verkhratsky, 2005). Stim1 and Stim2 are both suited to detect these small drops in ER Ca^2+^ levels and mediate SOCE in resting brain neurons. As a matter of fact, SOCE is the most proper route to redirect extracellular Ca^2+^ into the cytosol of non-firing neurons, as Ca^2+^ flux through Orai channels is tightly regulated by the electrochemical gradient across PM: at hyperpolarized membrane potentials, the driving-force sustaining Ca^2+^ inflow through Orai2 (i.e., the putative neuronal Orai isoform in mouse) is enhanced, thereby favoring resting Ca^2+^ entry and stimulating SOCE-dependent downstream targets. As described in the paragraph “Evidence about Stim- and Orai-mediated Ca^2+^ entry in brain neurons,” this mechanism is triggered by Stim2 (i.e., the hippocampal Stim isoform) in order to refill the ER Ca^2+^ store in cortical neurons (Berna-Erro et al., 2009) and sustain spine morphogenesis in mouse hippocampal neurons (Sun et al., 2014). Similarly, Stim1 (i.e., the cerebellar Stim isoform) and Orai2 interact to recharge the ER Ca^2+^ store in mouse Purkinje neurons (Hartmann et al., 2014). Accordingly, the genetic deletion of Stim1 and Orai2 depletes the ER Ca^2+^ pool at resting membrane potential (V_M_), thereby abrogating InsP_3_- and mGluR1-dependent intracellular Ca^2+^ release and impairing cerebellar motor behavior (Hartmann et al., 2014). It is presumable that resting SOCE maintains [Ca^2+^]_i_ and ER Ca^2+^ levels also in the hippocampus, but this hypothesis remains to be experimentally probed.

## SOCE Controls Gene Expression in Brain Neurons

Basal SOCE does not only modulate spinogenesis and ER Ca^2+^ levels; it also drives gene transcription in mouse cerebellar granule cells (Lalonde et al., 2014). Sp4 is a neuron transcription factor that governs the expression of multiple tissue-specific and housekeeping genes and is implicated in memory formation and behavioral processes relevant to psychiatric disorders (Zhou et al., 2005; Pinacho et al., 2011). Stim1 is activated in hyperpolarized (i.e., quiescent) granule cells by the partial depletion of the ER Ca^2+^ pool and relocates into sub-membranal *puncta* that are juxtaposed to both Orai1 and Orai2. The resulting SOCE triggers Sp4 ubiquitylation and proteasomal degradation, but does not stimulate cAMP response element-binding protein (CREB) phosphorylation. Moreover, membrane depolarization (i.e., synaptic activity) refills ER Ca^2+^ load, thereby dismantling Stim1 puncta, deactivating SOCE and, ultimately, restoring Sp4 abundance (Lalonde et al., 2014). This study did not examine which Orai isoform mediates SOCE, but Orai2 is the most likely candidate (Hartmann et al., 2014). Furthermore, future investigations will have to assess if this mechanism is deranged in schizophrenia, in which Sp4 down-regulation is associated to disease symptoms (Pinacho et al., 2011; Hooper et al., 2014). We should, however, point out that Stim1-dependent regulation of Sp4 represents a novel mode of excitation-transcription coupling in central neurons. Herein, Ca^2+^-dependent transcription factors, including CREB, downstream regulatory element antagonist modulator (DREAM), nuclear factor of activated T cells (NFATs) and nuclear factor-κb (NF-κB), are usually activated by membrane depolarization, rather than hyperpolarization (Hagenston and Bading, 2011). The presence of a basal SOCE endows neurons with two potentially distinct sources of Ca^2+^ to regulate gene expression in a timely manner: VOCCs and ROCs, which act during synaptic transmission and at depolarized V_M_, and SOCE, which occurs at resting V_M_ (**Figure 1**). We cannot rule out the possibility that other yet unknown transcription factors are selectively activated by the constitutive influx of Ca^2+^ via store-operated channels in brain neurons. This would permit them to activate or de-activate the expression of two distinct sets of genes depending on the extent of membrane excitation (i.e., synaptic activity).

## Evidence that SOCE Controls Neuronal Ca^**2+**^ Dynamics during Synaptic Excitation

Overall, available evidence indicates that Stim1 (in mouse cerebellum) and Stim2 (in mouse cortex and hippocampus) activate Orai2 to mediate SOCE in silent neurons to regulate spine morphogenesis, preserve ER Ca^2+^ levels and tune gene expression. However, SOCE could also play a role during neuronal excitation. Even a single synaptic stimulus fully depletes the ER Ca^2+^ pool in dendritic hippocampal spines (Emptage et al., 1999) and has, therefore, the potential to further stimulate Stim1 and Stim2 in firing neurons. Consistently, Stim1 was recently found to activate TRPC3 and mediate mGluR1-dependent slow excitatory post-synaptic potentials in mouse Purkinje neurons (Hartmann et al., 2014). Earlier work showed that SOCE contributes to elevate dendritic Ca^2+^ concentration during tetanic stimulation and participates to LTP generation at Schaffer collateral-CA1 synapses in hippocampal slices (Baba et al., 2003). Unfortunately, there are no studies in Stim- or Orai-deficient neurons to support this contention at molecular level. As aforementioned, Stim1 ablation prevents the Ca^2+^ response to synaptic stimulation in cerebellar Purkinje neurons, but this is due to previous depletion of the ER Ca^2+^ pool (Hartmann et al., 2014). If SOCE is basally activated to maintain ER Ca^2+^ concentration, it is very likely that the genetic disruption of its constituents will always depress Ca^2+^ transients independently on the role played by SOCE during the synaptic response. We predict that short-term incubations with specific Orai inhibitors could unveil whether and how SOCE modulates Ca^2+^ dynamics in firing neurons (for a list of selective blockers, see Parekh, 2010; Moccia et al., 2014a). SOCE could be relevant to dictate the polarity, i.e., LTD vs. LTP, of the changes in synaptic plasticity. For instance, low (bursts < 250 ms) and high frequency (bursts > 250 ms) mossy fiber discharge induce, respectively, LTD and LTP by activating two distinct patterns of post-synaptic Ca^2+^ signals in cerebellar granule cells. A low increase in [Ca^2+^]_i_ generated by VOCCs and NMDA receptors elicits LTD, while a sustained elevation in [Ca^2+^]_i_ associated to mGluR1 stimulation results in LTP (Gall et al., 2005). One might hypothesize that SOCE is selectively engaged during high, but not low, frequency transmission, due to the larger depletion of the ER Ca^2+^ pool. As a consequence, SOCE would participate to the increase in post-synaptic [Ca^2+^]_i_ that triggers the phosphorylation cascade culminating in LTP induction (Higley and Sabatini, 2012). This hypothesis is consistent with the physical coupling of Orai channels with their downstream Ca^2+^-sensitive decoders. For instance, Stim1-, Stim2-, and Orai1-dependent Ca^2+^ entry stimulate CaMKII and extracellular-signal regulated kinase (ERK), which are required for LTP expression and maintenance, respectively (Parekh, 2009; Voelkers et al., 2010; Lüscher and Malenka, 2012; Sun et al., 2014; Umemura et al., 2014). Moreover, SOCE could control spine extension not only in silent neurons, but also during synaptic stimulation. We predict that future investigation will provide more insights on the impact of Stim and Orai proteins on short- and long-term synaptic plasticity.

## Stim1 Interaction with Voltage-Operated Ca^**2+**^ Channels

Stim1 does not only associate with Orai1 and Orai2 (and TRPC3) in brain neurons. CaV1.2 (α1C) mediates L-type voltage-operated Ca^2+^ currents in cortex, hippocampus, cerebellum and neuroendocrine system (Cahalan, 2010). Recent work demonstrated that Stim1 regulates CaV1.2 expression and activity in rat cortical neurons (Harraz and Altier, 2014). Store depletion causes ER-resident Stim1 to relocate in close proximity to PM: herein, Stim1 CAD strongly interact with the COOH-terminus of CaV1.2, thereby attenuating L-type Ca^2+^ currents (Park et al., 2010). In the longer term, Stim1 causes CaV1.2 internalization and this process leads to the complete loss of functional CaV1.2 channels (Park et al., 2010). Similar results were reported in A7r5 vascular smooth muscle cells, albeit the acute effect of Stim1 on CaV1.2-mediated Ca^2+^ entry is remarkably stronger as compared to rat neurons. Furthermore, Stim1 is trapped by Orai1 nearby CaV1.2 channels only in A7r5 cells (Wang et al., 2010). Notably, this study assessed that Stim2 does not interact with CaV1.2 and does not suppress voltage-operated Ca^2+^ influx (Wang et al., 2010). More recently, Stim1 was found to physically interact also with CaV3.1 (α1G), which mediates T-type VOCCs and is widely expressed throughout the CNS (Cueni et al., 2009). Similar to CaV1.2, Stim1 prevents the surface expression of CaV1.3, thereby preventing any cytotoxic Ca^2+^ overload in contracting cells (Nguyen et al., 2013). It is still unknown whether this mechanism operates also in brain neurons; however, these data confer Stim1 the ability to finely tune Ca^2+^ entry through different membrane pathways, as it promotes Ca^2+^ inflow through Orai channels while blocks VOCCs. For instance, Stim1 activates the I_CRAC_ and fully inhibits VOCCs in Jurkat T cells (Park et al., 2010), in which it reaches higher levels of expression as compared to central neurons (Cahalan, 2010). The relatively low abundance of Stim1 in brain neurons might explain why it does not suppress voltage-operated Ca^2+^ influx in these cells. However, it might exert a profound impact on neuronal Ca^2+^ homeostasis. Based on the data reported so far, the following scenario may be predicted. Intense synaptic activity causes Stim1 to partially hinder VOCCs and activate Orai2 and Orai1 in mouse and rat neurons, respectively. This mechanism would enable Stim1 to: (1) trigger SOCE-dependent pathways involved in LTP induction and expression (see paragraph entitled “Evidence that SOCE controls neuronal Ca^2+^ dynamics during synaptic excitation”) and/or (2) limit voltage-dependent Ca^2+^ inflow, thereby preventing cytotoxic Ca^2+^ accumulation. This hypothesis makes physiological sense as Orais are low-conductance, Ca^2+^-selective channels tightly coupled to their decoders (Parekh, 2010), while VOCCs are high-conductance channels that generate global increases in [Ca^2+^]_i_ (Cueni et al., 2009; Catterall, 2011). At the same time, Stim1 interaction with CaV1.2 and CaV1.3 could help understanding Stim1 and Orai1 co-localization into puncta-like clusters upon ER depletion in mouse hippocampal and cortical neurons. Herein, Stim1 could reduce voltage-operated Ca^2+^ entry during synaptic activity by decreasing CaV1.2 and CaV1.3 activity with (CaV1.3) or without (CaV1.2) Orai1 contribution. This subtle regulation of Ca^2+^ influx could prevent detrimental Ca^2+^ entry into firing neurons and, therefore, it would be interesting to examine the interaction between Stim1 and VOCCs not only in healthy neurons, but also in the presence of neurodegenerative disorders.

## The Involvement of SOCE in Neurological Disorders

It is well-known that dendritic spines are eliminated or compromised during aging and neurodegenerative disorders, such as AD, thereby resulting in synaptic failure and memory loss (Bezprozvanny and Hiesinger, 2013; Popugaeva and Bezprozvanny, 2013, 2014). These events have been associated to the dysregulation of ER Ca^2+^ homeostasis: for instance, analysis of familial AD (FAD)-causing mutations in presenilins (*PSEN1* and *PSEN2* genes) has revealed an increase in ER Ca^2+^ concentration that leads to a compensatory increase in InsP_3_R and RyR expression and SOCE down-regulation (Bezprozvanny and Hiesinger, 2013; Popugaeva and Bezprozvanny, 2013, 2014). Indeed, SOCE has long been associated to FAD pathogenesis in both cortical and hippocampal neurons (Yoo et al., 2000; Ris et al., 2003); a recent study demonstrated that Stim2-SOCE-CaMKII pathway is impaired in hippocampal neurons isolated from the PS-1 M146V knock-in (KI) mouse model of FAD. Derangement of Stim2 signaling leads to mushroom spine loss (Sun et al., 2014), defective spatial learning (Berna-Erro et al., 2009) and has been identified in aging brain mice and sporadic AD human brains (Sun et al., 2014). Importantly, overexpression of Stim2 rescues both its downstream signaling cascade and dendritic spine morphology (Sun et al., 2014). Furthermore, a recent investigation showed that HEK cells stably over-expressing Stim1 and Orai1 display a drastic reduction in the generation and secretion of Aβ peptides (Zeiger et al., 2013). However, there are no data about their involvement in AD pathogenesis in murine models or human specimens of this disease, yet. Nevertheless, additional evidence suggests that Orai1, as well as Stim2, may be crucial for the pathogenesis of neurodegenerative diseases and in traumatic brain injury. Accordingly, Stim2 underpins the glutamate-induced cholesterol loss in rat hippocampus that features both acute neuronal injury or AD and Parkinson’s disease. Excessive glutamatergic neurotransmission induces a massive Stim2-dependent increase in post-synaptic spines that causes the enzyme cholesterol 24-hydroxylase (CYP46A1) to translocate from ER to PM and remove cholesterol (Sodero et al., 2012). Exaggerated glutamatergic stimulation may also deplete neurons of glutathione (GSH), thereby triggering a specific program of cell death termed oxytosis through an increase in reactive oxygen species (ROS) and a late phase of extracellular Ca^2+^ entry. A recent study further showed that ROS-induced Ca^2+^ influx in the mouse hippocampal cell line HT22 requires a functional Orai1, but not Stim1 or Stim2 (Henke et al., 2013). This result would make physiological sense as Orai1 does not seem to be regulated by any of the ER Ca^2+^ sensors in mouse hippocampus, but it has been clearly linked to oxidative stress in other cell types (Bogeski et al., 2010). Stim1 and Stim2 have also been implicated in neurological disorders: they are both up-regulated in dentate gyrus, CA1 and CA3 regions of chronic epileptic mice and in a hippocampal sample from a subject with medial temporal lobe epilepsy (Steinbeck et al., 2011). Moreover, 2-APB and ML-9, two rather non-selective SOCE inhibitors (Parekh, 2010; Moccia et al., 2014a), abolish interictal spikes and rhythmize epileptic burst activity in organotypic epileptic hippocampal slices (Steinbeck et al., 2011). This implies that SOCE stimulates neuronal excitability *per se* or by activating Ca^2+^-dependent depolarizing channels, such as Transient Receptor Potential Melastatin 4 (TRPM4) or TRPM5 (Guinamard et al., 2010). Therefore, these preliminary findings indicate that SOCE is altered in several major neural diseases in the man, thereby hinting at Stim and Orai proteins as novel targets to be probed in the quest of alternative treatments for neurological and neurodegenerative disorders.

## Conclusion

It has long been thought that excitable cells, including neurons, do not require SOCE to replenish their endogenous Ca^2+^ stores and regulate cell behavior (Putney, 2003). Nevertheless, it is now clear that Stim and Orai proteins are expressed in brain neurons and control a growing number of functions (**Figure 1**). We have the opportunity to witness the beginning of a new era in the study of neuronal Ca^2+^ dynamics. This is why only scarce preliminary information is currently available regarding the localization and pathophysiological roles served by the diverse Stim and Orai isoforms in central neurons.

First, there is a tissue- and species-dependent pattern of expression. In the mouse, which provides a multitude of transgenic models suited for the investigation of cognitive process in health and disease, Stim1 reaches the highest expression levels in the cerebellum, while Stim2 is far more abundant in the hippocampus. This is consistent with preliminary findings implicating Stim1 in the control of motor coordination (Hartmann et al., 2014) and Stim2 in memory acquisition and storage (Berna-Erro et al., 2009; Sun et al., 2014).

Second, both Stim1 (cerebellum) and Stim2 (cortex and hippocampus) trigger SOCE in mouse central neurons even in the absence of synaptic activity (**Table 4**). This feature is surprising when considering that Stim2, but not Stim1, activates Ca^2+^ inflow in response to mild-store depletion in other cell types. It turns out that Stim2 should activate basal SOCE in cerebellum as well. However, Stim1 is far more abundant in this region than in other brain areas. Moreover, the ER becomes rapidly depleted of Ca^2+^ in the absence of Ca^2+^ influx in mouse cerebellar granule cells (Lalonde et al., 2014): this suggests that ER Ca^2+^ levels are very low in these cells and might, therefore, rapidly reach the threshold for Stim1 activation. The use of genetic indicators of ER Ca^2+^ concentration will be useful to assess whether [Ca^2+^]_ER_ differs between mouse cerebellum (supposed to be lower due to the constitutive activation of Stim1) and cortex/hippocampus (supposed to be higher due to the constitutive activation of Stim2). The situation is clearer in rat neurons, in which Stim1 and Stim2 accomplish two distinct roles: Stim2 gates resting Ca^2+^ entry, whereas Stim1 elicits Ca^2+^ inflow in response to larger ER depletion (**Table 4**).

Third, there is no evidence that Orai1 mediates SOCE in mouse brain neurons, in which Orai2 stands out as the most likely candidate to gate Ca^2+^ in response to ER emptying (**Table 4**). This issue gains more relevance when considering that no clear-cut role has hitherto been attributed to this isoform in any cell type (Hoth and Niemeyer, 2013). However, it will be necessary to confirm this hypothesis in Orai2-deficient neurons isolated from brain regions other than the cerebellum (Hartmann et al., 2014), such as cortex and hippocampus. Conversely, Orai1 seems to form the store-operated channel pore in rat neurons (Gruszczynska-Biegala et al., 2011). One further question arises as to the role played by Stim1 and Orai1 in mouse cortex. As illustrated in the paragraph entitled “Evidence about Stim- and Orai-mediated Ca^2+^ entry in brain neurons,” Stim1 and Orai1 co-localize in response to ER depletion, but do not mediate SOCE, in mouse cortical and hippocampal neurons. We suggest that Stim1 and Orai1 fulfill alternative functions in these cells. This hypothesis is corroborated by several evidences illustrated throughout the text. For instance, Orai1 could be recruited by Stim1 into discrete *puncta* in order to be activated by an additional stimulus, such as oxidative stress (Henke et al., 2013). On the other hand, Stim1 does not only activate Orai1: it also regulates the expression and activity of CaV1.2 and CaV3.1 and associates with several members of the TRPC family (Lee et al., 2010), including TRPC3 (Hartmann et al., 2014). Furthermore, Orai1 and Orai3 were recently shown to control cell proliferation independently of Ca^2+^ entry in HEK293 cells; this unexpected finding was explained by proposing that an integral element of the channel protein harbors an enzyme domain or acts as a scaffold for other signaling molecules (Borowiec et al., 2014). Therefore, the interactome of Stim1 and Orai1 should be carefully evaluated in order to obtain key insights on how such protein controls neuronal processes (Munaron, 2014).

Fourth, despite the fact that Stim1, Stim2, Orai1, and Orai2 were shown to mediate SOCE in brain neurons, no study has attempted to measure I_CRAC_ in these cells. Recording I_CRAC_ is a demanding challenge due to the tiny conductance (in the fS range) of the underlying channel, which generates sub-pA currents in the whole-cell mode of patch-clamp and often fall below the resolving power of current amplifiers (Beech, 2009). This task could become even more arduous in neurons which express a complex battery of high conductances (in the pS range) VOCs and ROCs that should be fully inhibited before recording such an exceedingly small current. Nevertheless, addressing the biophysical features of I_CRAC_ in naïve neurons (for instance, in *ex vivo* brain slices) could confirm the notion that Orai2 and Orai1 mediate SOCE, respectively, in mouse and rat by exploiting their electrophysiological differences (**Table 1**).

We foresee that future work will unveil new yet undiscovered aspects of the pathophysiological role fulfilled by Stim and Orai proteins in central neurons. For instance, SOCE amplitude is dramatically enhanced in cerebellar granule neurons obtained from cellular prion protein (PRP^c^)-KO mice (Lazzari et al., 2011) and in HD medium spiny striatal neurons (MSNs; Wu et al., 2011); however, the role of Stim and Orai proteins has not been evaluated in these models. Nevertheless, there are enough data available to predict that these proteins will provide the molecular target to devise alternative therapies of life-threatening neurodegenerative disorders. Exciting developments are expected in the field: future research will certainly dissect the role of Stim and Orai proteins in the pathophysiological regulation of neuronal Ca^2+^ homeostasis and excitability.

## Conflict of Interest Statement

The authors declare that the research was conducted in the absence of any commercial or financial relationships that could be construed as a potential conflict of interest.
